# Low-Cost National Media-Based Surveillance System for Public Health Events, Bangladesh 

**DOI:** 10.3201/eid2204.150330

**Published:** 2016-04

**Authors:** Trong T. Ao, Mahmudur Rahman, Farhana Haque, Apurba Chakraborty, M. Jahangir Hossain, Sabbir Haider, A.S.M. Alamgir, Jeremy Sobel, Stephen P. Luby, Emily S. Gurley

**Affiliations:** Centers for Disease Control and Prevention, Atlanta, Georgia, USA (T.T. Ao, J. Sobel, S.P. Luby);; Institute of Epidemiology, Disease Control and Research, Dhaka, Bangladesh (M. Rahman, F. Haque, A. Chakraborty, S. Haider, A.S.M. Alamgir);; icddr,b, Dhaka (F. Haque, A. Chakraborty, M.J. Hossain, S.P. Luby, E.S. Gurley)

**Keywords:** disease outbreaks, surveillance, infectious diseases, public health, newspapers, television, Bangladesh

## Abstract

We assessed a media-based public health surveillance system in Bangladesh during 2010–2011. The system is a highly effective, low-cost, locally appropriate, and sustainable outbreak detection tool that could be used in other low-income, resource-poor settings to meet the capacity for surveillance outlined in the International Health Regulations 2005.

The International Health Regulations (IHR) 2005 state that an effective public health system should conduct surveillance activities to enhance detection, reporting, notification, verification, response, and collaboration in the event of an outbreak (*1*).Traditional approaches to disease surveillance heavily rely on laboratories, points of care, or population-based surveillance for disease reporting. Although these approaches are generally effective, traditional surveillance systems are limited by reporting delays, high cost, and reliance on an overburdened healthcare infrastructure for information collection.

In 2009 the Institute of Epidemiology, Disease Control and Research (IEDCR) of Bangladesh, the national agency responsible for disease surveillance and outbreak investigation, established a surveillance system to monitor print and television media to quickly detect outbreaks. We describe this media-based surveillance system and report results from a formal evaluation of its characteristics.

## The Study

IEDCR contracted for a media scanning company to identify relevant news stories for this surveillance system (Figure). Each day, the company collected the major daily newspapers available in the capital city of Dhaka shortly after morning distribution. Trained staff members at the company read through each paper to identify any health-related article and scan them into PDF file format. Other staff members scanned television news reports and recorded relevant video clips.

**Figure:**
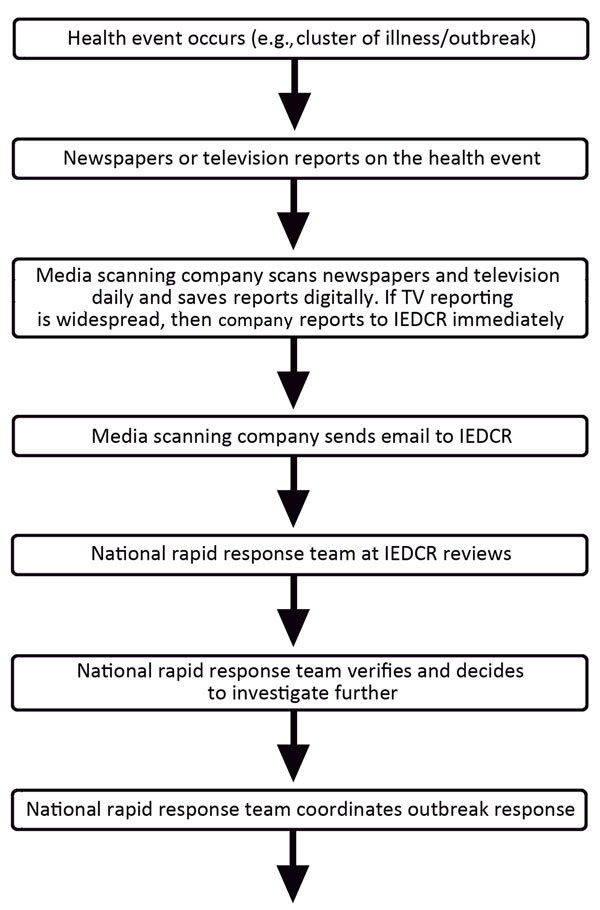
Information flow for national media-based public health surveillance system, Bangladesh. IEDCR, Institute of Epidemiology, Disease Control and Research.

The national rapid response team, consisting of key staff members from IEDCR, received a daily email containing all identified health-related newspaper articles and video clips. The team examined each news item and decided whether it warranted an outbreak response on the basis of expert clinical and epidemiologic knowledge; public health importance (e.g., number of cases and deaths reported, severity of symptoms); and verification by local health officials. For the purposes of this analysis, IEDCR retrospectively created a database of reported events sent by the media scanning company, which included the number of reported events, outbreak etiology, news source, and the outcome of each investigation. The outbreaks reported were classified by media type, etiology, and season.

To assess the surveillance system’s performance, we followed the guidelines for evaluating public health surveillance set by the US Centers for Disease Control and Prevention (CDC) (*2*).We aimed to assess all 9 recommended system qualities: simplicity, flexibility, data quality, acceptability, sensitivity, positive predictive value (PPV), representativeness, timeliness, and stability (*3*). However, because we did not have external data to serve as a reference standard or comparator, we were unable to assess the sensitivity and PPV of the system. We calculated the proportion of all outbreak investigations conducted by IEDCR that were first detected through the media surveillance. We interviewed key stakeholders, including the manager and staff of the contracted media scanning company, the director of IEDCR, and national rapid response team members. We also conducted a group discussion with 4 journalists representing large national newspapers to explore the processes of obtaining information for health events and timeline of reporting.

A total of 36 news sources were scanned regularly in this media surveillance system: 23 (64%) were Bengali language newspapers, 6 (17%) were English language newspapers, and 7 (19%) were Bengali language television channels. From May 2010 through September 2011, the media scanning company captured and delivered 2,821 news stories to IEDCR. Of those, 2,501 (89%) were health related, 810 items included the term “outbreak” (29% of total), and 196 were the first reports of a possible outbreak (7%); 90% of all outbreak reports came from Bengali language media sources.

During the same period, the national rapid response team investigated 30 outbreaks, 21 (70%) of which were first detected through this surveillance system. At the rate of US$125/year to hire the scanning company, the total cost for the 16-month period was US$167. The cost of contracting with the scanning company for each outbreak detected with this system was therefore approximately US$8 (US$167 divided by 21 outbreaks). Outbreaks of diarrhea, measles, and anthrax were reported through this system (Table 1). Outbreaks were reported year-round and from 51 of the 64 administrative districts in Bangladesh (Table 2).

**Table 1:** Outbreaks first identified by event-based surveillance and investigated by IEDCR, Bangladesh, May 2010–September 2011*

Outbreak no.	Date	Etiology reported by media	Confirmed etiology
1	2010 May	Anthrax	Cutaneous anthrax
2	2010 Jun	Anthrax	Cutaneous anthrax
3	2010 Jul	Unknown poisoning	Unintentional pesticide poisoning
4	2010 Jul	Mass psychogenic illness	Mass psychogenic illness
5	2010 Jul	Suspected pneumonia	Bronchiolitis
6	2010 Jul	Unknown animal scratch	Rabies
7	2010 Jul	Food poisoning	Food poisoning
8	2010 Jul	Diarrhea	Diarrhea
9	2010 Aug	Cutaneous anthrax	Cutaneous anthrax
10	2010 Nov	Diarrhea	Cholera
11	2010 Nov	Suspected high-energy biscuit poisoning	Mass psychogenic illness after biscuit consumption
12	2010 Dec	Suspected pneumonia	Bronchiolitis
13	2010 Dec	Suspected rabies	Rabies
14	2011 Apr	Diarrhea	Cholera
15	2011 May	Cutaneous anthrax	Cutaneous anthrax (5 outbreaks)
16	2011 Jun	Cutaneous anthrax	Cutaneous anthrax (2 outbreaks)
17†	2011 Jun	Unusual duck and geese mortality†	Avian influenza, subtype H5N1, in geese, but no human cases detected
18	2011 Jul	Cutaneous anthrax	Cutaneous anthrax
19	2011 Jul	Cutaneous anthrax	Cutaneous anthrax
20	2011 Aug	Unknown disease	Influenza B virus infection
21	2011 Aug	Cutaneous anthrax	Cutaneous anthrax

*IEDCR, Institute of Epidemiology, Disease Control and Research, Bangladesh. †Non–human-related outbreak.

**Table 2:** Case numbers for media-reported outbreaks, by cause and season, Bangladesh, May 2010–September 2011

Reported etiology	No. (%) cases				
	Pre-monsoon, Mar–May	Monsoon, Jun–Sep	Post-monsoon, Oct–Nov	Winter, Dec–Feb	Total
Diarrhea	24 (50)	7 (15)	10 (21)	7 (15)	48 (100)
Anthrax	3 (14)	19 (86)	0	0	22 (100)
Mass psychogenic illness	9 (45)	10 (50)	0	1 (5)	20 (100)
Upper respiratory infection	0	2 (33)	3 (50)	1 (17)	6 (100)
Measles	3 (60)	0	0	2 (40)	5 (100)
Other/unknown	31 (33)	49 (52)	5 (5)	10 (11)	95 (100)
Total	70 (36)	87 (44)	18 (9)	21 (11)	196 (100)

Key informant interviews consistently indicated that the system was simple, flexible, timely, and acceptable because it used existing media infrastructure and required only minimal costs to contract with a company to compile daily reports of news items. Changes to the system could be implemented effectively through frequent communications between the media scanning company and IEDCR. The system was widely acceptable by all stakeholders and was considered a valuable component of disease surveillance in Bangladesh.

We were unable to quantitatively assess the coverage of remote areas by national newspapers, especially those lacking easy access to telecommunication infrastructure. The system moderately captured a representative sample of possible sources of information in the country. Both Bengali- and English-language news items were collected, although only newspapers available in Dhaka were included, so some newspapers with only local circulation were not available for review. 

Timeliness and stability of the system were both high. Although time from outbreak onset to reporting in the media source might vary, once media sources learned of an outbreak, publication occurred within 24 hours. Because it was low-cost, low-tech, and highly acceptable to all stakeholders, the system was highly stable.

This media surveillance identified outbreaks of emerging infections that might not have been otherwise investigated, including several outbreaks that were potential public health events of international concern. In the context of global health security, international donors should support media-based surveillance to further strengthen existing traditional indicator-based approaches. 

The media-based surveillance system in Bangladesh fills a gap that is not covered by other global event-based surveillance systems, which collect publically available information about potential health threats mostly from Internet sources (*4*), such as ProMED (*5*), BioCaster, and HealthMap (*6*). Although these systems collect and analyze enormous amounts of information from the Internet regarding potential health threats, they are limited by the inability to process information in local, non-English languages or to capture information not on the Internet. In 2013, ≈341 million persons in the world (5%) spoke English as a first language (http://www.nationsonline.org/oneworld/most_spoken_languages.htm); clearly, most news sources in the world are written in languages other than English and, therefore, are beyond the reach of these English language event-based surveillance systems.

Although all stakeholders were knowledgeable about their duties and responsibilities and the procedures which needed to be followed, no written standard operating procedures were in place by which we could evaluate process performance. Written procedures, for both the media scanning company and IEDCR staff, could enhance system performance by fostering sustainability and ensuring standardization.

Our evaluation of this surveillance system was limited in 2 key ways. Because the database for the system was created retrospectively over a short period, some reports may have been missing from the database. In addition, the absence of other data sources meant that we were unable to determine the sensitivity and PPV of the system. However, neither of these limitations influences the high proportion of outbreaks detected through this system nor the low cost per outbreak detected, the most critical findings from our evaluation.

## Conclusions

IEDCR in Bangladesh has created an innovative, low-cost, locally appropriate solution for event-based surveillance that helps to meet the purposes of the country’s surveillance goals and IHR requirements. This surveillance system could serve as a model for outbreak detection in other resource-poor countries.
